# Characteristics of paediatric burn injuries seen in the tertiary emergency centre, South Africa

**DOI:** 10.4102/safp.v67i1.6009

**Published:** 2025-01-08

**Authors:** Ntsovelo Mugwena, Rule Human, Maria M. Geyser

**Affiliations:** 1 Department of Family Medicine, Division of Emergency Medicine, Faculty of Health Sciences, University of Pretoria, Pretoria, South Africa; 2 Department of Family Medicine, Division of Emergency Medicine, Faculty of Health Sciences, Kalafong Provincial Tertiary Hospital, Pretoria, South Africa

**Keywords:** paediatric burns, South Africa, burn wound care, referral patterns, burn characteristics, burns outcome

## Abstract

**Background:**

Burn injuries cause significant morbidity and mortality, with prevalence in developing countries such as South Africa. This study aimed to determine the characteristics and referral patterns of burn injuries.

**Methods:**

A retrospective observational study was conducted in a single emergency centre, Kalafong Provincial Tertiary Hospital, from 01 January 2021 to 31 December 2021. The study included patients < 13 years with burn injuries.

**Results:**

A total of 266 patients were identified. Males (*n* = 144, 54.1%) had a higher prevalence of incurring burn injuries. The majority of injuries were secondary to scald burns (*n* = 237, 89.1%). A total of 208 (78.2%) patients had a percentage of total body surface area (%TBSA) of < 10%, and 257 (96.6%) had superficial partial-thickness burns. Only 77 (28.9%) cases were from referral centres and there was no relationship between referral pattern and %TBSA. Majority (*n* = 248, 93.2%) received no pre-hospital wound care. Only 108 (40.6%) patients were admitted and the median length of hospital stay (interquartile range [IQR]) was 7 days (2 to 9). There was a significant relationship between the length of hospital stay and %TBSA burns (*p* < 0.001).

**Conclusion:**

The pattern of burn injuries in patients is similar to previous studies carried out predominantly in townships in South Africa. Most referrals were found to be appropriate and complied with institutional burn injury admission protocol, although pre-hospital wound care was inadequate.

**Contribution:**

Primary burn injury care is vital to reduce morbidity and mortality, and development of programmes for public awareness of burn injuries remains crucial.

## Introduction

A burn injury is damage to tissue layers because of fire, electricity, scalds, chemicals, and radiation.^1^ The prevalence of burn injuries varies by geographical setting, socioeconomic status, ethnicity, cultural practices, age, and gender.^2^ It continues to cause significant morbidity and mortality in developing countries.^3,4^

The World Health Organization (WHO) has estimated that yearly 310 000 burn-related deaths occur globally. The overall mortality rate of paediatric burn injuries is low, but it is seven times higher in developing countries than in developed countries.^2,5^

In sub-Saharan Africa, between 18 000 and 30 000 child-related burns occur annually.^1^ In South Africa, burns are the third most common cause of morbidity and mortality among patients < 18 years.^6^ Research worldwide has found that patients < 5 years are at higher risk of burn injuries^3,7,8^; that scald injuries are the most common mechanism of injury^2,3,5,6,9,10^; and that burn injuries are more prevalent in male patients than in female patients.^3,7,10,11^

The risk of wound contamination increases with inappropriate pre-hospital wound care.^12^ Cool water irrigation decreases the depth of burn injury, speeds up healing and decreases the incidence of intensive care unit admission. Although there is no consensus on the duration of irrigation, the longest time recorded was 20 min.^12^

Pre-hospital interventions from previous studies included applying running water, ice, egg, oil, toothpaste, and traditional medicine.^1,13^ The substances used included *Aloe vera* and cassava paste (Nigeria), chalk and sunlight soap (South Africa [SA]), burnt snail shells and tomato juice (Ghana), and urine, fur, glycerine, lotion, and milk (Tanzania).^14^

Certain burn cases carry significant mortality and morbidity which require hospital admission and appropriate management. According to the American Burn Association, these specialised cases include all burn patients aged < 1 year, patients aged between 1 and 2 years with burns > 5% total body surface area (TBSA), third-degree burns, partial-thickness burns > 10% TBSA, and burns on special areas such as the face, hands, feet, genitalia, perineum, or major joints.^15^

Epidemiological studies performed in SA elaborated on burn injuries occurring in rural and urban areas of the Western Cape and KwaZulu-Natal.^6,10,11,13^ This study aimed to determine the characteristics and referral patterns of burn injuries seen at Kalafong Hospital, which serves the surrounding townships.

The objective was to quantify and describe characteristics of paediatric burn injuries, referencing the mechanism and extent of injury, the referral patterns from other healthcare centres, duration taken to seek care in health centre and any relevant pre-hospital wound care provided.

## Research methods and design

This was a retrospective observational study, conducted in Kalafong Provincial Tertiary Hospital emergency centre, which renders service to residents mainly in the townships of Tshwane.

### Inclusion criteria

- All patients < 13 years presenting with burn injuries in the emergency centre from 01 January 2021 to 31 December 2021.
- Both primary presentation and referrals.

### Exclusion criteria

- All patients with files that could not be traced by the record department.

All consecutive subjects with burn injuries were included for the period 01 January 2021 to 31 December 2021. Although the statistician calculated 128 cases to be the minimum required sample size for the study, 266 cases were identified. All files were traced and analysed.

### Data collection and analysis

Data were collected retrospectively by identifying patients from the paediatric register in the emergency centre. Files were obtained from the record department and data were captured using a data collection sheet.

Data included demographics (age and gender), mechanism and setting of injury, time between burn incidence and presentation to hospital, primary presentation or referral, level of referral facility, pre-hospital wound care, %TBSA and depth of burn, disposition, length of hospital stay, and accidental versus non-accidental burn injuries.

The data were captured onto Microsoft Excel spreadsheets and analysed using Statistical Package for Social Sciences (SPSS) version 26 software. Associations between categorical variables were tested by using the Chi-square test. The Pearson’s correlation test was used to determine the association between interval variables, for example, length of stay, depth of injury, and %TBSA burns.

### Ethical considerations

Ethical clearance was obtained from the University of Pretoria Health Sciences Research Ethics Committee (No: 455/2022), and permission from the Kalafong Provincial Tertiary Hospital management was obtained. Personal details of patients were omitted during data collection to maintain confidentiality of information kept in clinical records.

## Results

A total of 266 patients were identified. Male patients (*n* = 144, 54.1%) had a higher prevalence of incurring burn injuries than female patients (*n* = 122, 45.9%) (Table 1). The ages ranged between 5 months and 13 years, with a predominance in age 1 year to less than 3 years (*n* = 143, 53.8%) (Table 1).

**TABLE 1:** Demographics and characteristics of burn injuries.

Demographics and characteristics variable	Category	Frequency (*n*)	Percentage
Gender	Female	122	45.9
	Male	144	54.1
Age	Infant (< 1 year)	42	15.8
	Toddler (1 year to < 3 years)	143	53.8
	Preschooler (3 year to < 6 years)	31	11.7
	Scholar: 6–13 years	50	18.8
Cause of injury	Accidental	263	98.9
	Non-accidental	3	1.1
Setting	Household	260	91.7
	Public	6	2.3
Mechanism of injury	Scalds	237	89.1
	Fire	14	5.3
	Electrical	11	4.1
	Hot surface contact	4	1.5
%TBSA burns	< 10%	208	78.2
	10% – 20%	46	17.3
	> 20%	12	4.5
Depth of burn injury	Superficial partial thickness	257	96.6
	Deep partial thickness	8	3
	Full thickness	1	0.4
Presentation time	< 24 h	244	91.7
	24–48 h	4	1.5
	49–72 h	4	1.5
	> 72 h	14	5.3
Primary presentation (P) or referral	P	189	71.1
	R	77	28.9

P, primary presentation; R, referral.

Most of the injuries (*n* = 260, 91.7%) occurred at home, and only six (2.3%) were in public spaces. It was further noted that majority of the cases seen were accidental injuries, with only three cases associated with non-accidental events (Table 1).

By far the most burn injuries (*n* = 237, 89.1%) were caused by scalds. Fourteen (5.3%) were caused by fire, 11 (4.1%) by electricity, and four (1.5%) by hot surface contact (Table 1). Most scald injuries were seen in toddlers. No chemical burns were documented during the study period. There was statistical significant correlation between the mechanism of burn injury and %TBSA (χ^2^ = 26.396, *p* = 0.003). Furthermore, there was significant correlation between mechanism of injury and depth of burn injury (χ^2^ 77.491, *p* < 0.001).

Most patients (*n* = 187, 70.3%) were primary presentations, whereas 44 (16.5%) were referred from community health centres, 15 (5.6%) from district hospitals and 20 (7.5%) from other tertiary hospitals.

Of the patients who presented to the hospital primarily, 167 (88.4%) had %TBSA burns < 10%, 18 (9.5%) between 10% and 20% TBSA, and four (2.1%) had > 20% TBSA burns.

**FIGURE 1:**
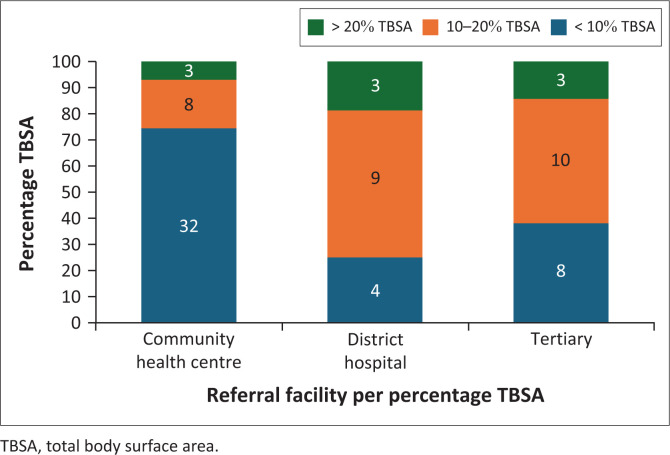
Referral patterns by percentage TBSA.

In referral patients, 41 patients (53.2%) had burns < 10% TBSA, of which majority were referred from community health centres, whereas 27 (35.1%) had burns between 10% and 20% TBSA and 9 (11.7%) had > 20% TBSA burns. There was no relationship between the two variables (Figure 1).

Most of the patients sustained superficial partial-thickness burns. Of these, 183 (68.8%) presented primarily and 74 (27.8%) were referred. There was no association between the two variables.

Eight patients (3%) had deep partial-thickness burns, whereas only 1 (0.4%) patient had full thickness burns secondary to an electrical burn injury from a residential high-voltage transformer (Table 1). The majority of superficial partial-thickness burns (*n* = 233, 90.6%) were secondary to scalds; 12 (4.7%) were fire related, and the remaining 12 (4.7%) were electricity-related. There were no significant statistical associations between the demographic variables and %TBSA burns and depth of burn injury.

**FIGURE 2:**
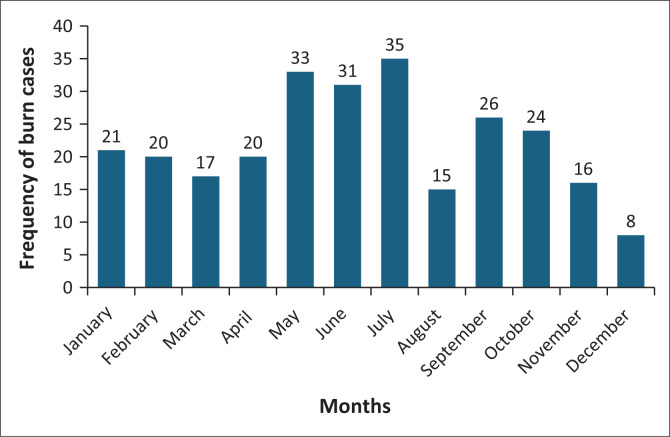
Months in which burns were incurred.

Figure 2 shows that most injuries occurred during the colder months of May (*n* = 33, 12.4%), June (*n* = 31, 11.7%), July (*n* = 35, 13.2%), and December with the least presentation (*n* = 8, 3%).

The majority of cases (*n* = 244, 91.7%) presented in less than 24 h, 4 (1.5%) between 24 h and 48 h, while 18 (6.8%) presented after 48 h. Although there was high number of early hospital presentation, majority (*n* = 248, 93.2%) did not receive pre-hospital wound care, while only four (1.5%) received water irrigation of unknown duration (Table 2).

**TABLE 2:** Pre-hospital wound care.

Pre-hospital intervention	Frequency (*n*)	Percentage
None	248	93.2
Toothpaste	5	1.9
Betadine ointment	1	0.4
Burnshield	4	1.5
Toothpaste, egg	1	0.4
Wound cream	1	0.4
Water/irrigation	4	1.5
Burn gauze	1	0.4
Paraffin gauze	1	0.4

**Total**	**266**	**100.0**

The disposition of patients included outpatient management and admission to general ward or intensive care unit (ICU). Only 108 (40.6%) patients were admitted. Among admitted patients, nine (8.3%) were admitted to the ICU and 99 (91.7%) to a general ward. Although admission indications were not clearly stated, a trend towards certain case presentation and admission toward or ICU were noticed (Table 3).

**TABLE 3:** Indication for admission in general ward and intensive care unit.

Ward admission	ICU admission
Low-voltage electrical burns Facial burns (for airway and feeding monitoring) Other specialised burn sites (genitals, hands, joint involvement and circumferential burns) Minor septic wounds Non-accidental injuries (for child protection service intervention)	High-voltage electrical burns with secondary injuries including: traumatic brain injury, crush injury, cardiac dysrhythmia Facial injuries with associated airway compromise requiring intubation Septic wounds, complicated with septic shock

ICU, intensive care unit.

**FIGURE 3:**
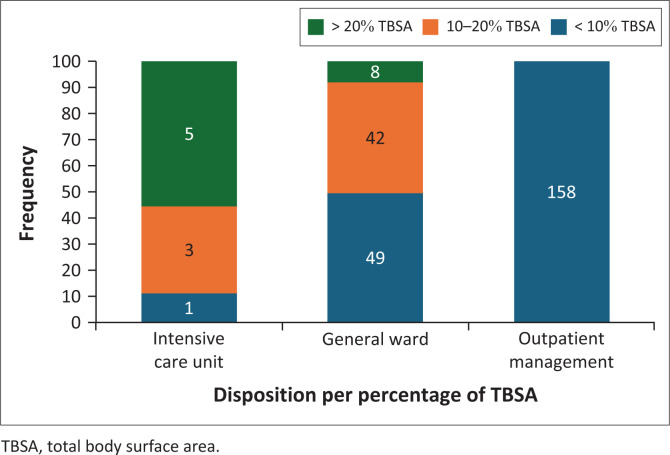
Disposition per %TBSA.

With regard to the distribution of admitted patients per %TBSA: overall, 50 patients (46.3%) had %TBSA burns < 10%, 45 (41.7%) had %TBSA burns between 10% and 20%, and 13 (12%) had %TBSA burns > 20% (Figure 3). All patients with %TBSA burns > 10% were admitted, of which majority were admitted to the general ward.

Among patients admitted, 100 (92.6%) had superficial partial-thickness burns, seven (7.4%) had deep partial-thickness burns and only one (1%) had full thickness burns. All patients managed as outpatients had superficial partial-thickness burns and < 10% TBSA burns. Although there was significant association between the disposition and %TBSA burns (χ^2^ = 85.076, *p* < 0.001), there was no significant association found between depth of burn injury and disposition.

The median length of hospital stay (IQR) was 7 days (2 to 9). An association was found between length of hospital stay and depth of burn injury (Pearson’s *r*-value: 0.346, *p* < 0.001), and length of hospital stay and %TBSA burns (Pearson’s *r*-value: 0.57, *p* < 0.001).

**FIGURE 4:**
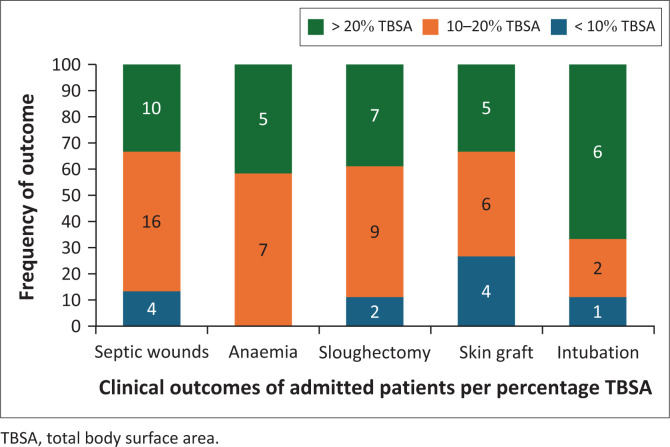
Clinical outcomes of admitted patients.

In Figure 4, it is illustrated that majority of patients with complications and requiring surgical intervention were patients admitted with > 10% TBSA burn injuries. Only one mortality case was reported, which occurred in the emergency centre. A 3-year-old male patient from home presented more than 72 h post-scald burn injury with < 10% TBSA burns (involving lower limbs and genitals). On arrival, the patient already had septic burn wounds, hypovolemic shock, and anaemia.

## Discussion

This study aimed to provide overall characteristics and referral patterns of burn injuries in the paediatric population in Tshwane, South Africa. Burn injuries were more prevalent among males than females. Similar results were shared by previous studies conducted worldwide. Zhou et al. emphasised that although incidences of burns were higher in rural than urban areas, male patients were still at higher risk of sustaining burns in both areas.^3^ A Nigerian and an Ethiopian study showed different results, reporting a higher instance of burn cases in female patients, which they attributed to cultural practices.^1,2^

Children under 5 years continue to be more at risk of sustaining burns because of their impulsiveness, a lack of awareness, general curiosity, and total dependency on caregivers.^3,6,7,8^ The most common mechanism of burn injury was scalds (hot milk exposure, pulling of hot liquid containers, and steam during traditional practices). Similar trends were noticed in previous studies carried out in SA, Nigeria, China and India.^2,3,5,6,9,10^ Some countries reported more cases of fire-related burn injuries,^4,9^ especially in rural areas still dependent on fire as energy source for cooking. According to the WHO, fire burns are related to gender because of household roles mostly filled by young females.^5^ The majority of these burn injuries occurred at home where the vulnerable paediatric population is mostly found.

Most patients sustained burns with < 10% TBSA, reflecting similar results to those of Scheven et al. in their KwaZulu-Natal study,^13^ although other studies showed a higher percentage of patients with > 10% TBSA burns.^2^ Superficial thickness burns were prevalent; yet some of these cases still required hospital admission mainly because of the area of burns (face, genitals, joint areas) or mechanism of injury, such as electrical burns. Among referrals, reasons for referral included > 10% TBSA burns, involvement of vital areas (face, genitals and joints), and mechanism of injury, specifically electricity.

This study showed a higher rate of burns during winter because of re-warming practices. Burn injuries can also be attributed to cultural practices, such as the Harmattan season in Nigeria when the dry period increases the incidence of burnt material and prolonged cooking periods,^4^ and festivals that include the lighting of firecrackers.^3^

The safety of patients is paramount, and it remains the healthcare workers’ obligation to act upon any concerns of child abuse. Most cases were accidental, as also reported by Gessesse et al.^1^ Non-accidental injuries encountered were reported to the relevant authorities.

Public education campaigns emphasising burn prevention strategies and first aid treatment are important. This study focused on assessing characteristics of burn, presentation time to emergency centre, and pre-hospital wound care. The majority of adults responded quickly, with over 93.2% presenting to hospital in less than 24 h.

In the literature, water irrigation was the most common form of pre-hospital wound care.^14^ In Ethiopia, the use of traditional medicine was prevalent.^1^ An Australian study showed that 33.1% of cases received adequate pre-hospital cool running water.^16^ Yet in this study, over 94% of patients did not receive any pre-hospital wound care, only 1.6% irrigated with water, and the duration was unspecified. The use of toothpaste, non-specified wound ointment, and burnshield dressing, was also recorded. This indicates the importance of educating the public about burns and appropriate burn injury wound care to decrease morbidity and mortality of burn injuries.

All patients with > 10% TBSA and burn depth other than superficial partial-thickness burns were admitted to the hospital. According to Hollander et al., in their paediatric group the length of hospital stay was 1 day/%TBSA for all burn percentages and the length of hospital stay was influenced by the %TBSA and the depth of burn injury.^6^ This study also showed a significant association between these parameters, suggesting that a higher %TBSA burns increases the chance of prolonged hospital stay.

Complications, such as dehydration, sepsis, contractures, and mortality rate also increase with significant %TBSA burns, requiring a multidisciplinary approach to manage, and longer time to recover. Some patients required surgical intervention such as debridement and skin graft. The incidence thereof increased with burn depth that exceeded superficial partial-thickness area.

During the study period, there was only one recorded mortality case: a patient who sustained < 10% TBSA from scald injuries and had a delayed hospital presentation. Studies have recorded an increase in mortality rate with an increase in %TBSA affected.^6^

### Limitations of study

This was a retrospective observational study that was conducted in a single centre. A larger multicentre study with inclusion of non-township dwellers will aid in a wider assessment of burn injury prevalence and other possible contributing factors.

## Conclusion and recommendations

This study found that male patients are more at risk of developing burn injury, with predominance in patients under 3 years. The common aetiology was scalding from pulling hot liquid containers and tripping over low-lying hot-water buckets. These are preventable incidences, and safety measures are important to minimise the prevalence of burn injuries. Of the patients observed, most sustained superficial thickness burns with morbidity lower than for full thickness burns.

Many factors may contribute to the extent of burns including presentation time to the emergency centre and pre-hospital wound care. Although the majority of patients in this study were brought to the emergency centre in less than 24 h, most of them did not receive pre-hospital wound care.

Child safety is a priority and caregivers’ involvement in prevention strategies is important. The public must be educated in pre-hospital wound care to prevent aggravation of burn injuries. This can be accomplished through community teaching programmes.

Burn referral protocols should be readily available at community healthcare centres and district hospitals to aid in appropriate referrals to tertiary hospitals.

## References

[1] Gessesse FG, Yitayew YA. Epidemiology of burn injury among children’s attended Felege Hiwot Referral Hospital in Bahir Dar town, Amhara regional state, Ethiopia, 2017. J Pediatr Neonatal Care. 2020;10(1):21–27. 10.15406/jpnc.2020.10.00408

[2] Olawoye OA, Iyun AO, Ademola SA, Michael AI, Oluwatosin OM. Demographic characteristics and prognostic indicators of childhood burn in a developing country. Burns. 2014;40(8):1794–1798. 10.1016/j.burns.2014.04.008 24933574

[3] Zhou B, Zhou X, Ouyang LZ, et al. An epidemiological analysis of paediatric burns in urban and rural areas in south central China. Burns. 2014;40(1):150–156. 10.1016/j.burns.2013.04.020 23747041

[4] Isiguzo C, Opara C, Nnadozie U, Opara K. Burn injury in tertiary health facility in South East Nigeria: A 2 year prospective study. Burns Open. 2020;4(4):153–157. 10.1016/j.burnso.2020.08.001

[5] WHO. Burns [homepage on the Internet]. [updated 2018 Mar 06; cited 2023 May 22]. Available from: https://www.who.int/news-room/fact-sheets/detail/burns

[6] Den Hollander D, Albert M, Stran A, Hardcastle TC. Epidemiology and referral patterns of burns admitted to the burns centre at Inkosi Albert Luthuli Central Hospital, Durban. Burns. 2014;40(6):1201–1208. 24439933 10.1016/j.burns.2013.12.018

[7] Puthumana JS, Ngaage LM, Borrelli MR, Rada EM, Caffrey J, Rasko Y. Risk factors for cooking-related burn injuries in patients, WHO Global Burn Registry. Bull World Health Organ. 2021;99(6):439–445. 10.2471/BLT.20.279786 34108754 PMC8164180

[8] Dhopte A, Tiwari VK, Patel P, Bamal R. Epidemiology of pediatric burns and future prevention strategies – A study of 475 patients from a high-volume burn centre in North India. Burns Trauma. 2017;5:1. 10.1186/s41038-016-0067-3 28164140 PMC5286678

[9] Forbinake NA, Dongmo G, Ohandza CS, Chichom-Mefire A, Fokam P, Beyiha G. Epidemiologic and clinical profile of burns in a tertiary hospital in sub-Saharan Africa. Burns Open. 2019;4(1):22–7.

[10] Van Niekerk A, Rode H, Laflamme L. Incidence and patterns of childhood burn injuries in the Western Cape, South Africa. Burns. 2004;30(4):341–347. 10.1016/j.burns.2003.12.014 15145192

[11] Parbhoo A, Louw QA, Grimmer-Somers K. Burn prevention programs for patients in developing countries require urgent attention: A targeted literature review. Burns. 2010;36(2):164–175. 10.1016/j.burns.2009.06.215 19854000

[12] Mclure M, Macneil F, Wood FM. A rapid review of burns first aid guidelines: Is there consistency across international guidelines? Cureus. 2021;13(6):e15779. 10.7759/cureus.15779 34295589 PMC8291991

[13] Scheven D, Barker P, Govindasamy J. Burns in rural Kwa-Zulu Natal: Epidemiology and the need for community health education. Burns. 2012;38(8):1224–1230. 10.1016/j.burns.2012.04.001 22698838

[14] Outwater AH, Van Braekel T. Prehospital care of burn injuries in Africa: A review, 1990–2018. Burns. 2020;46(8):1737–1745. 10.1016/j.burns.2019.08.009 31785926

[15] Bettencourt P, Romanowski KS, Joe V, et al. Updating the burn centre referral criteria: Results from the 2018 eDelphi consensus study. J Burn Care Res. 2020;41(5):1052–1062. 10.1093/jbcr/iraa038 32123911 PMC7510842

[16] Frear CC, Griffin B, Kimble R. Adequacy of cool running water first aid by healthcare professionals in the treatment of paediatric burns: A cross-sectional study of 4537 children. Emerg Med Australas. 2021;33(4):615–622. 10.1111/1742-6723.13686 33191592 PMC9292905

